# White matter microstructure of children with sensory over-responsivity is associated with affective behavior

**DOI:** 10.1186/s11689-023-09513-w

**Published:** 2024-01-02

**Authors:** Jamie Wren-Jarvis, Rachel Powers, Maia C. Lazerwitz, Jaclyn Xiao, Lanya T. Cai, Hannah L. Choi, Annie Brandes-Aitken, Robyn Chu, Kaitlyn J. Trimarchi, Rafael D. Garcia, Mikaela A. Rowe, Mary C. Steele, Elysa J. Marco, Pratik Mukherjee

**Affiliations:** 1 Department of Radiology & Biomedical Imaging, University of California, UCSF, 185 Berry St, Suite 350, Box 0946, San Francisco, CA 94143-0946 USA; 2 Cortica Healthcare, 4000 Civic Center Dr., Suite 100, San Rafael, CA 94903 USA; 3 Department of Psychology and Neuroscience, University of Colorado Boulder, Boulder, CO USA; 4 Lifetime Neurodevelopmental Care Center, San Rafael, CA USA

**Keywords:** Sensory over-responsivity, Sensory processing disorder, DTI, MRI, White matter, Somatization, Depression

## Abstract

**Background:**

Sensory processing dysfunction (SPD) is linked to altered white matter (WM) microstructure in school-age children. Sensory over-responsivity (SOR), a form of SPD, affects at least 2.5% of all children and has substantial deleterious impact on learning and mental health. However, SOR has not been well studied using microstructural imaging such as diffusion MRI (dMRI). Since SOR involves hypersensitivity to external stimuli, we test the hypothesis that children with SOR require compensatory neuroplasticity in the form of superior WM microstructural integrity to protect against internalizing behavior, leaving those with impaired WM microstructure vulnerable to somatization and depression.

**Methods:**

Children ages 8–12 years old with neurodevelopmental concerns were assessed for SOR using a comprehensive structured clinical evaluation, the Sensory Processing 3 Dimensions Assessment, and underwent 3 Tesla MRI with multishell multiband dMRI. Tract-based spatial statistics was used to measure diffusion tensor imaging (DTI) and neurite orientation dispersion and density imaging (NODDI) metrics from global WM and nineteen selected WM tracts. Correlations of DTI and NODDI measures with measures of somatization and emotional disturbance from the Behavioral Assessment System for Children, 3rd edition (BASC-3), were computed in the SOR group and in matched children with neurodevelopmental concerns but not SOR.

**Results:**

Global WM fractional anisotropy (FA) is negatively correlated with somatization and with emotional disturbance in the SOR group but not the non-SOR group. Also observed in children with SOR are positive correlations of radial diffusivity (RD) and free water fraction (FISO) with somatization and, in most cases, emotional disturbance. These effects are significant in boys with SOR, whereas the study is underpowered for girls. The most affected white matter are medial lemniscus and internal capsule sensory tracts, although effects of SOR are observed in many cerebral, cerebellar, and brainstem tracts.

**Conclusion:**

White matter microstructure is related to affective behavior in children with SOR.

**Supplementary Information:**

The online version contains supplementary material available at 10.1186/s11689-023-09513-w.

## Background

Sensory processing dysfunction (SPD), broadly defined, refers to a clinical deficit in the ability to modulate, discriminate, or create an organized response to sensory information and affects up to 16% of children [1]. Children born prematurely and those with congenital malformations affecting white matter tracts such as agenesis of the corpus callosum are particularly vulnerable to SPD [2–6]. Due to the disruptions in sensory processing, children with SPD may demonstrate atypical or delayed intellectual, language, or motor milestones [7]. Based on a wide variety of criteria and measures, SPD is commonly associated with other conditions, including co-occurrence in up to 70% of children with autism spectrum (ASD), anxiety, attention deficit/hyperactivity (ADHD), and developmental coordination disorders [1, 7–13]. While sensory processing differences, particularly those related to auditory and visual discrimination, have been recognized and studied in the context of autism for decades, the importance of sensory over-responsivity (SOR), a component of SPD, has only recently gained widespread neuroscience and community attention [14–18]. SOR is characterized by extreme negative reactions to innocuous, common sensory experiences and is frequently reported in the auditory and tactile domains, therefore making these domains an excellent starting point for exploration of neural mechanisms which can then be expanded to visual, gustatory/olfactory, vestibular, and proprioception domains.

### Sensory over-responsivity is a public health concern

SOR is conservatively estimated to affect 2.5% of children in community samples and has substantial deleterious impact on learning and function at home, at school, and in the community [19–21]. In toddlers with ASD, SOR explained 39–45% of parental stress and family impairment, independent of ASD symptom severity [22]. Thus, the toll of SOR on the individual, parents, siblings, and school community is tremendous. There is a gap, however, in recognition and research into SOR due to the historical conception that SOR is “behavioral”, secondary to ASD, or more egregiously due to “bad parenting” [23, 24]. Furthermore, there is a concern that SOR or SPD is not a “recognized disorder” and thus may not warrant study. However, with neuroimaging findings codifying differences in children with SPD and emerging reports of genetic etiologies [25–28], it is clear that, whatever term we use to describe it, be it dysfunction, disorder, or condition—sensory processing needs further study. By pairing detailed phenotyping with advanced neuroimaging, this project can further our understanding of SOR as a “brain-based” condition that, just like headaches or seizures, can be understood and improved with treatment.

### Mapping the white matter microstructural basis of sensory behavior using diffusion MRI

Neuroimaging analyses, both functional and structural, have primarily taken a categorical diagnostic and statistical manual (DSM) approach rather than a sensory domain approach. However, pivotal work ties sensory processing to higher order behavioral function [29]. Functional imaging data from Green et al. suggests that youths with co-morbid ASD and SOR are over-responsive to mildly aversive sensory stimuli and show slower neural habituation in the amygdala and sensory cortex to these stimuli [30, 31]. Furthermore, during sensory stimulation, children with ASD show reduced modulation of pulvinar functional connectivity with the cortex but increased connectivity with subcortical areas, including the amygdala. This is posited to play a role in maintaining attention and affective responses to the sensory stimuli [19]. Using diffusion tensor imaging (DTI) structural neuroimaging, our lab reported that children ages 8–12 years with broadly defined SPD, but not ASD, show white matter microstructure differences compared to matched typically developing controls—specifically decreased white matter (WM) microstructural integrity measured as reduced fractional anisotropy (FA) in posterior cerebral tracts involving sensory cortex [32] as well as the cerebellar peduncles which are involved in corticocerebellar circuits crucial for sensory processing [33]. One limitation of these and most other prior studies of SPD is that diagnosis was conducted using a caregiver questionnaire, the Sensory Profile [34], rather than the structured clinical assessment proposed in this work. Also, there were no attempts in these prior imaging studies to subtype SPD by important categories of behavior such as SOR, sensory underresponsivity or sensory seeking.

### Direct phenotypic assessment of SOR advances investigation of neural networks

While most parent report measures do quantify individual sensory domains (i.e., auditory, visual, tactile), they conflate aspects of sensory processing: modulation, discrimination, and sensory-based motor ability. This complex phenotype may benefit group differentiation (SPD or Neurotypical), but it is not optimal for mapping brain-behavior networks. Thus, even sensory modulation must be subdivided into sensory over-responsivity, under-responsivity, and seeking. For example, in the auditory domain of the Sensory Profile [34], a widespread research tool, parents are asked if their child “holds hands over ears to protect ears from sound.” This is an indicator of over-responsivity. On the other hand, parents are also asked whether the child “enjoy[s] strange noises/seeks to make noise for noise’s sake” and “doesn’t respond when name is called but you know the child’s hearing is OK.” These questions probe auditory seeking and under-responsivity, respectively. These queries, and five others, are combined in the auditory processing subscale, an important start but not specific enough for brain-behavior correlation.

In our previous work, we compared in-lab assessment of auditory discrimination using the Acoustic Index of the Differential Screening Test for Processing (DSTP) with the more complex parent-report composite of auditory processing from the Sensory Profile. We found that in-lab sensory assessment shows more continuous mapping of both chidren with SPD (but not ASD) and typically developing controls with the FA of relevant posterior sensory WM tracts from DTI [25]. This correlational analysis is not intended to make a clinical diagnosis or to achieve a discriminative diagnosis, but rather to detect which white matter connections contribute meaningfully to specific sensory processes. Children can then be sub-grouped (high/low) on that specific function in order to tease out the network contributions using connectome analysis. Here, we characterize the neural mechanisms of SOR versus other presentations of SPD.

As the majority of ASD and other neurodevelopmental research has focused on auditory and visual discrimination or use of parent report measures, there is a gap in our direct assessment of SOR. We address this gap by assessing a community acquired cohort of children with neurodevelopment concerns using a direct assessment measure—the Sensory Processing 3 Dimensions: Assessment (SP3D:A), described in detail in the methods section. The SP3D:A measures three primary constructs or dimensions: (1) sensory modulation, (2) sensory discrimination, and (3) sensory-based motor abilities. It is organized by sensory domains (auditory, visual, tactile, proprioceptive, and vestibular), and each domain has multiple subtests. Mulligan et al. [26] reports significant SPD versus neurotypical discriminative validity using the modulation assessment in children 4 to 13 years of age. In addition, there was strong correlation between the SP3D:A direct assessment of auditory and tactile modulation with the relevant scales of the Sensory Profile, a parent report tool. The internal consistency of the auditory SOR subtests is 0.76 (range: 0.66–0.86) and the tactile SOR subtests is 0.87 (range: 0.77–0.94). Inter-rater reliability is 67% for both auditory and tactile atypical modulation behavior and 98% and 92%, respectively, for auditory and tactile typical modulation behavior [27]. In collaboration with the Sensory Processing Disorders Workgroup, iterative versions of the SP3D:A have been administered to 176 children with neurodevelopmental concerns and neurotypical controls to phenotype auditory and tactile SOR [35]. Pairing SOR assessment with advanced neuroimaging is the next step.

### Does white matter microstructure affect affective behavior in children with SOR?

Given the aforementioned sensory, attentional, thalamic, and limbic features of SOR, we hypothesize that school-age children with SOR will exhibit altered WM microstructure that is related to affective behavior. Since SOR involves hypersensitivity to external stimuli, we specifically posit that children with SOR may exhibit compensatory neuroplasticity in the form of superior white matter microstructural integrity that is protective against internalizing behavior such as somatization and its consequent emotional disturbances including generalized unhappiness and depression. Therefore, children with SOR that have reduced white matter microstructural integrity are postulated to have greater levels of somatization, as found in somatic symptoms disorder, and related emotional disturbances. This hypothesis is tested using matched school-age children with neurodevelopmental concerns but not SOR as the “non-SOR” control group.

We test this hypothesis regarding SOR with DTI for comparison with existing literature on SPD, ASD and ADHD, as well as with more advanced neurite orientation dispersion and density imaging (NODDI) analysis using multi-shell diffusion MRI (dMRI) at higher diffusion-weighting factors than is typical for DTI [36, 37]. Low FA and axial diffusivity (AD) and high mean diffusivity (MD) and radial diffusivity (RD) are DTI markers of reduced white matter microstructural integrity. However, they do not provide insight into the biophysical basis of white matter microstructural changes. NODDI characterizes white matter microstructure in terms of its intracellular volume fraction, which serves as a neurite density index (NDI), its fiber orientation dispersion index (ODI) that measures the coherence of white matter tracts, as well as its free water fraction (FISO). Higher NDI typically reflects more developed microstructure with superior integrity, whereas higher ODI and FISO are both thought to represent less developed microstructure with poorer integrity [36, 38, 39]. Although NODDI is increasingly being utilized in investigations of individuals with autism, this is one of the first applications of dMRI biophysical compartment modeling approaches such as NODDI to the study of children with sensory over-responsivity who do not have co-morbid ASD, including a recent pilot study suggesting alterations in the hemispheric lateralization of both DTI and NODDI metrics in children with SOR versus non-SOR controls [40].

Neuroimaging evidence supporting the hypothesis that SOR is linked to affective behavior would aid brain-based biomarker development to better stratify risk for adverse mental health outcomes in children, a major and growing unmet public health need.

## Methods

### Participants

Children ages 8–12 years old who presented to a community neurodevelopmental clinic or by community referral were evaluated for study eligibility based on a standardized neurodevelopmental parent report form reviewed by the study coordinator (MAR, MCL, RP). All participants were recruited from the clinic located in Marin County, California, which at the time of the study only took privatized insurance. The primary inclusion criteria of the neurodevelopmental concern cohort were determined using the early symptomatic syndromes eliciting neurodevelopmental clinical examinations-questionnaire (ESSENSE-Q-REV; Supplementary Fig. 1), a 12-question caregiver screener for ESSENSE disorders including ASD, ADHD, language impairments, developmental coordination disorder, and Tourette’s syndrome [41]. No participants in this study showed complete neurotypical developmental history. Based on previous reports, children whose parents marked at least one “yes” or two “maybe/a little” answers were highly likely to meet criteria for an ESSENCE disorder and thus eligible for this study focused on understanding SOR in the context of clinical practice. Children meeting research criteria for ASD were excluded to focus on the wider understudied population of children with SPD without autism. An ASD designation was assigned to participants that scored above the ASD diagnostic cutoff on both a caregiver report form, the Social Communication Questionnaire (SCQ) [42], and the Autism Diagnostic Observation Schedule, Second Edition (ADOS-2) [43]. Children with a nonverbal index ≤ 70 on the Wechsler Intelligence Scale for Children (Fifth Edition) were excluded from the study to ensure the ability to complete cognitive tasks and cooperation in the scanner environment, thereby lowering the probability of motion artifacts. Further exclusion criteria for this study include caregiver(s) unable to complete intake forms which were in English, in-utero toxin exposure, gestational age < 32 weeks or intrauterine growth restriction (birth weight < 1500 g), hearing or visual impairment limiting the ability to participate in assessment, active epilepsy, malignancy, or known or suspected brain injury/malformation. The Behavioral Assessment System for Children, 3rd edition (BASC-3), was used to measure affective behavior, including somatization and, more generally, emotional disturbance. Clinical concerns arising after review of the neurodevelopmental parent report form were adjudicated by the study pediatric neurologist (EJM) and pediatric neuroradiologist (PM).

### Sensory measurement

A direct assessment of sensory characterization was performed by a licensed pediatric occupational therapist using the SP3D:A [44]. The direct assessment SP3D:A was used to distinguish typical sensory individuals from those with atypical sensory processing. Those with atypical processing could show sensory craving, sensory under-responsivity, and/or sensory over-responsivity for each activity. We focused on sensory over-responsivity for this analysis. The assessment was used to determine the prevalence of AOR, tactile over-responsivity (TOR), and visual over-responsivity (VOR) of children with neurodevelopmental concerns. Three auditory, four tactile, and three visual probes from the SP3D:A were utilized to determine the SOR categorization. The three auditory measures are “Sounds and Pictures Matching Game” which has the participant listen to 10 tracks matching the sounds played to pictures on page; “Orchestra Time” which has the participant mimic the clapping of the examiner with three different instruments: cymbals, stick and symbol, and whistle; and “Find a Picture Game” which has the participant find a pictures in booklet while audio is played as background noise. The four tactile measures are “Goo Game” which has the participant manually scoop out a plastic dinosaur from a container of slime and “Painting Game” (3 parts) which has the participant use a paintbrush and foot scrubber to go up and down their own arm (wrist to shoulder) three times, then the participant uses a disposable foam oral swab to circle outside of their lips. The three visual measures: “Round and Round Game” where the participant watches a spinning black and white swirl disc for 20 s while examiner counts then instructs participant to stop looking and stare at a blank wall, “Lighting storm game” in which the participant moves 5 animals from in front of a strobe light to another location, and “Sparkle Game” in which the participant watches an electronic sparkle wheel for 20 s while the examiner counts. The games were scored 1 (typical), 2 (mild/moderate), or 3 (severe) in regard to the intensity of their aversive reaction to each game. Over-responsivity was determined with a score of 2 or 3 in any game of the respective sensory domain. Participants can be designated with sensory over-responsivity in one or multiple sensory domains. If a participant received a score above 10 for over-responsivity, they would be deemed SOR.

### MRI acquisition

All participants were scanned on a single Siemens 3 Tesla (3T) Prisma MRI scanner (Erlangen, Germany) using a 64-channel head coil. During scanning, participants viewed video entertainment of their choice via an MR-compatible audiovisual system. Whole brain dMRI was acquired at diffusion-weighting strengths (shells) of *b* = 1000 s/mm^2^ (64 diffusion-encoding directions) and 2500 s/mm^2^ (96 diffusion-encoding directions), with 5 *b* = 0 s/mm^2^ volumes per shell (TE = 72.20 ms, TR = 2420 ms, flip angle = 85°, slice thickness = 2.0 mm, in-plane resolution 2.0 mm) using single-shot spin echo echo-planar imaging. Two additional *b* = 0 s/mm^2^ volumes were acquired with forward and reverse phase encoding directions to be used for distortion correction. Simultaneous multiband (MB) excitation was used (MB factor = 3). The duration of diffusion scanning totaled 8 min. To prepare participants, their families were sent a cartoon of a child going into the scanner and a sound file of scanner sounds to play for the child before the scan. The day of the scan, children were given ample time to acclimate in the room and feel comfortable around the scanner prior to entering.

### Diffusion MRI processing

Each participant’s dMRI data underwent quality control inspections and the same processing pipeline to compute DTI and NODDI metrics. The FMRIB Software Library (FSL) version 6.0.2 (Oxford, UK) was used for imaging processing and DTI parameter computation. All diffusion scans were visually inspected for scanner and motion artifacts and excluded post-eddy processing if scans were calculated to have a high percentage of outlier replacements. The pair of forward and reverse phase-encoding images were used in FSL’s *topup* [45] to estimate susceptibility-induced off-resonance fields. The *b* = 1000 s/mm^2^ and *b* = 2500 s/mm^2^ scans were then concatenated. A brain mask was created from the first volume of the multi-shell data using Freesurfer’s SynthStrip [46]. FSL’s *eddy* was applied to the raw multi-shell diffusion data to correct for motion and eddy distortions, outlier replacement, susceptibility-by-movement, and slice-to-volume correction [47–50]. A second brain mask was created from the first volume of the *eddy* corrected data and applied for skull stripping. FSL’s automated quality control framework [51] was applied to the *eddy* corrected data to help determine within- and between-subject outliers. The *b* = 1000 s/mm^2^ shell was extracted from the processed multi-shell data and used to calculate DTI parameters. To increase SNR, the *b* = 0 s/mm^2^ volumes were averaged together and used as the first volume followed by the remaining 64 diffusion-weighted volumes; this input was used in FSL’s *dtifit* to calculate FA, MD, AD and RD maps. The processed, multi-shell data including the* b* = 3000 s/mm^2^ shell was utilized in the Accelerated Microstructure Imaging via Convex Optimization (AMICO) Toolbox [37] to calculate the NODDI metric maps including NDI, ODI and FISO.

### Statistical analysis

Tract-Based Spatial Statistics (TBSS) in FSL [52] was used to skeletonize and register the diffusion metric maps of each participant in order to perform region of interest (ROI) measurements along the white matter skeleton using the Johns Hopkins University (JHU) ICBM-DTI-81 White-Matter Labeled Atlas [53]. Using TBSS, “the most representative subject” was determined from the FA maps of all participants and used as the target image, as recommended for populations of young children. The target image was affine-aligned into MNI152 standard space. Each FA map was transformed by combining the non-linear transform to the target FA image and the affine transform from the determined target image to MNI152 space and resampled to 1 mm resolution. The registered FA maps were then averaged and thinned to generate a mean FA skeleton to represent the core of all white matter tracts. The FA white matter skeleton was thresholded to FA > 0.2 to exclude voxels containing gray matter and partial volume effects. Next, each subject’s FA data was projected onto this mean FA skeleton to get individual skeletonized FA maps. The skeleton voxels were filled with values from the nearest relevant tract center by searching perpendicular to the local skeleton structure for the maximum value in the FA image of the subject. Each participant’s MD, AD, RD, NDI, ODI, and FISO maps were then registered and projected onto the white matter skeleton to create skeletonized maps of each diffusion metric. Eleven major JHU ICBM-DTI-81 white matter pathways were included in the exploratory regional WM analysis, eight of which come in left- and right-sided pairs for a total of 19 individual tracts. These 11 pathways consist of commissural tracts of the corpus callosum: genu (GCC), body (BCC), and splenium (SCC); projection fibers of the internal capsule: anterior limb (ALIC) and posterior limb (PLIC) as well as the corona radiata: anterior (ACR) and superior (SCR); association fibers of the external capsule (EC) and the superior longitudinal fasciculus (SLF); and finally rhomboencephalic tracts of the brainstem and cerebellum, specifically the somatosensory projection fibers of the medial lemniscus (ML) and the outflow projection fibers of the superior cerebellar peduncle (SCP). The ROIs were calculated by taking the average voxel intensity of the skeletonized diffusion metric map within the binary mask of each white matter tract from the JHU ICBM-DTI-81 Atlas for all diffusion metrics. Global white matter values were calculated for each participant by taking the average voxel intensity within the entire skeletonized map of the whole brain for all diffusion metrics.

Unpaired homoscedastic two tailed *t* tests were used to compare group differences in average global and tract ROI diffusion metrics between SOR and non-SOR groups as well as their sex-specific subgroups. A false discovery rate (FDR) [54] adjustment was made on the *p* values to correct for multiple comparisons of the JHU white matter tract ROIs within each metric as well as global white matter within group correlations. BASC-3 raw scores and dMRI parameter values were normalized into *z* scores prior to running correlations. *T* statistics, correlations, and other descriptive statistics including mean, standard deviation, and Cohen’s *d* effect size were performed with Python v3.7.6 [55] statistical packages.

## Results

### Demographics

A total of 136 participants with neurodevelopmental concerns (age: x̅ = 10.17 years, SD ± 1.65; sex: 36 females/100 males) participated in cognitive and sensory testing as well as an MRI scan. Twenty-two participants were excluded for meeting ASD research criteria. After visual and post hoc outlier inspections of the remaining 114 children, 106 total participants were included in the imaging analysis after exclusions for poor quality of the multi-shell dMRI (*n* = 7) and inadequate behavioral assessments (*n* = 1). Of the 106 participants 74% of their parents identified as white, 8% as Asian, 17% as more than one race, and 1% preferred not to answer; 7% of the participant’s parents identified as Hispanic or Latin American, 89% as neither Hispanic nor Latin American, and 4% preferred not to answer. Further demographic information can be seen in Table 1 for the final number of included subjects, average age, and breakdown of intelligence scores within each group. There were no significant differences in age between any groups.

**Table 1:** Demographic breakdown between groups

**Group**	***n***	**Age** (*μ* ± σ)	**SP3D:A**	**WISC V**					
			**Over-responsivity sum score** (*μ* ± σ)	**FSIQ** (*μ* ± σ)	**FRI** (*μ* ± σ)	**PSI** (μ ± σ)	**VCI** (μ ± σ)	**VSI** (μ ± σ)	**WMI** (μ ± σ)
SOR	54	10.15 ± 1.66	12.17 ± 1.74	105.48 ± 14.98	108.94 ± 13.79	91.61 ± 14.97	109.28 ± 15.98	107.19 ± 13.22	99.76 ± 15.67
M-SOR	36	10.00 ± 1.58	12.26 ± 2.02	105.31 ± 15.04	108.86 ± 15.41	89.00 ± 13.99	108.89 ± 15.85	108.19 ± 12.89	101.00 ± 15.64
F-SOR	18	10.45 ± 1.83	12.00 ± 1.00	105.83 ± 15.29	109.11 ± 10.18	96.83 ± 15.90	110.06 ± 16.68	105.17 ± 14.01	97.28 ± 15.87
non-SOR	52	10.12 ± 1.66	10.00 ± 0.00	106.29 ± 12.70	107.23 ± 13.18	92.92 ± 12.36	111.42 ± 13.87	112.06 ± 12.45	102.17 ± 14.74
M-non-SOR	39	10.16 ± 1.61	10.00 ± 0.00	106.92 ± 12.13	108.08 ± 11.76	91.95 ± 12.82	112.05 ± 14.58	113.74 ± 11.89	102.28 ± 15.23
F-non-SOR	13	10.00 ± 1.84	10.00 ± 0.00	104.38 ± 14.63	104.69 ± 17.04	95.85 ± 10.81	109.54 ± 11.77	107.00 ± 13.21	101.85 ± 13.73
AOR	26	10.13 ± 1.59	11.96 ± 1.30	104.77 ± 16.68	106.08 ± 14.82	90.50 ± 15.96	111.58 ± 16.07	105.42 ± 13.38	99.50 ± 16.64
M-AOR	15	9.54 ± 1.22	11.93 ± 1.44	103.33 ± 16.19	104.53 ± 17.18	85.27 ± 14.34	112.07 ± 13.11	104.73 ± 11.13	99.67 ± 16.24
F-AOR	11	10.93 ± 1.72	12.00 ± 1.12	106.73 ± 17.92	108.18 ± 11.31	97.64 ± 15.88	110.91 ± 20.09	106.36 ± 16.50	99.27 ± 17.97

The number of subjects (*n*), as well as the average age, and standard deviation, SP3D:A over-responsivity score, and WISC-V score are reported for each main group and subsequent groups categorized by sex. Groups include subjects with neurodevelopmental concern subsequently divided into those with sensory over-responsivity (SOR), without sensory over-responsivity (non-SOR), and those with auditory over-responsivity only (AOR). Sex-specific groups are named with the “M” and “F” prefixes for boys and girls, respectively. *SP3D:A* Sensory Processing 3 Dimensions: Assessment WISC Wechsler Intelligence Scale for Children, *FSIQ* full-scale IQ, *FRI* Fluid Reasoning Index, *PSI* Processing Speed Index, *VCI* Verbal Comprehension Index, *VSI* Visual-Spatial Index, *WMI* Working Memory Index.

Processing Speed Index from the WISC-V is reduced in children with broadly defined SPD, including those with SOR; however, Full-Scale IQ is in the normal range for this cohort. Of the WISC-V scores, only Visuospatial Index differs between the SOR and non-SOR groups, being lower for children with SOR (*p* < 0.05).

### Global white matter analysis: SOR vs non-SOR and relationship to affective behavior

**Fig. 1:**
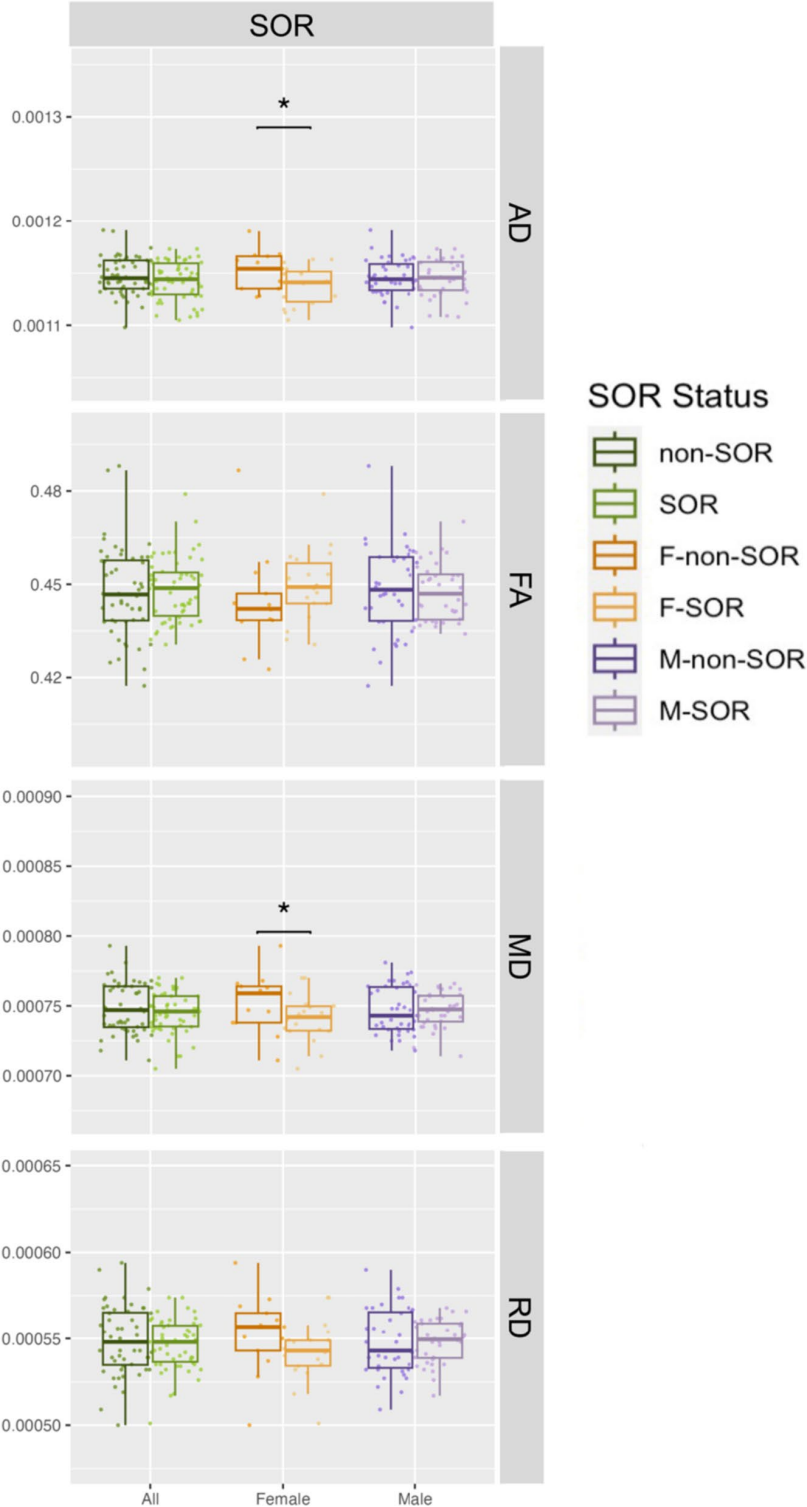
Global white matter DTI metrics: SOR versus non-SOR group comparisons. Comparisons for the SOR and non-SOR groups in solid green, the M-SOR and M-non-SOR groups in solid purple and the F-SOR and F-non-SOR groups in solid yellow. AD, MD, and RD values are in mm^2^/s. Statistically significant differences (*p* < 0.05) are marked with brackets and an asterisk above the comparison groups

**Fig. 2:**
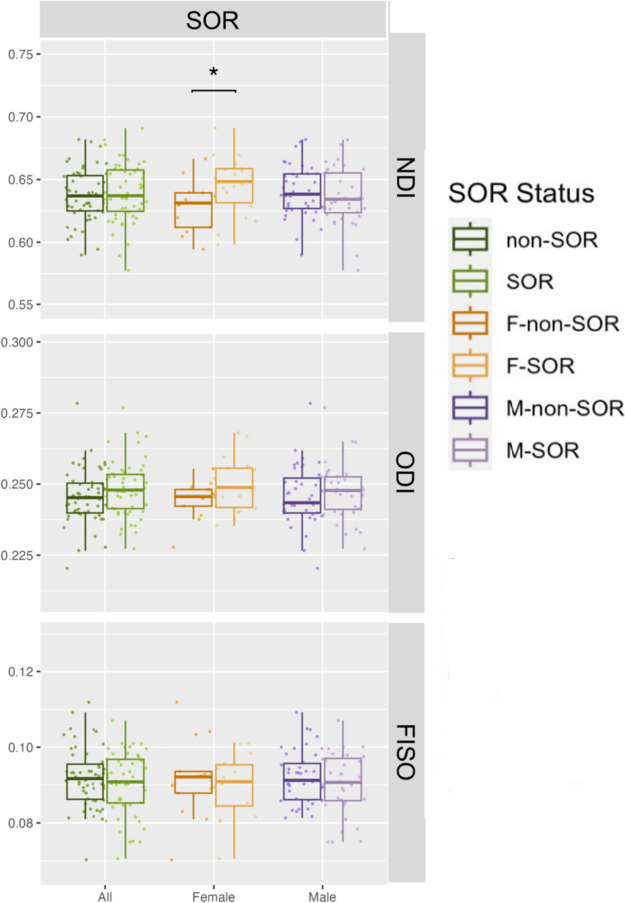
Global white matter NODDI metrics: SOR versus non-SOR group comparisons. Comparisons for the SOR and non-SOR groups in solid green, the M-SOR and M-non-SOR groups in solid purple and the F-SOR and F-non-SOR groups in solid yellow. Statistically significant differences (*p* < 0.05) are marked with brackets and an asterisk above the comparison groups

For general sensory over-responsivity, F-SOR have lower global AD (Cohen’s *d* =  − 0.843, *p* = 0.029) and higher global NDI (Cohen’s *d* = 0.924, *p* = 0.019) than the F-non-SOR group. No significant differences in global white matter are found between the combined male and female SOR and non-SOR cohorts or the M-SOR and M-non-SOR group on any diffusion metric. Global white matter differences of DTI and NODDI measurements between groups are illustrated in Figs. 1 and 2, respectively. Statistically significant decreases of AD and MD, but increases of NDI, were found in girls with SOR compared to girls without SOR. BASC-3 somatization raw scores are lower in the SOR group (6.02 ± 5.43) than the non-SOR group (6.73 ± 5.45), although this difference is not statistically significant (*p* = 0.53). There is also no difference in the emotional disturbance index category 4 (EDI4) sum scores between SOR (111.6 ± 20.9) and non-SOR (110.9 ± 21.5) groups (*p* = 0.88).

**Fig. 3:**
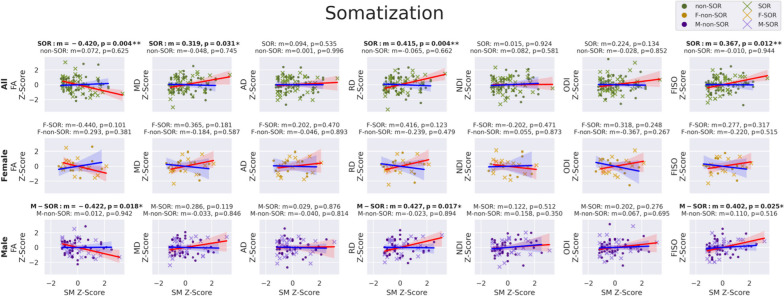
Correlation of DTI metrics (FA, MD, AD, & RD) and NODDI metrics (NDI, ODI, & FISO) in whole-brain global white matter with BASC-3 raw scores of somatization in the SOR and non-SOR groups of school-age children. Metric “*m*” is the slope of the linear regression and color shading around the regression line represents the 95% confidence interval. Boldface text indicates a significant correlation, with one asterisk for *p* < 0.05 uncorrected and two asterisks for FDR-corrected *p* < 0.05. SOR subjects are represented with "*x*" and non-SOR subjects with dots. All-subject comparisons are shown in green, female comparison in yellow, and male comparisons in purple. The regression line is marked red for SOR groups and blue for non-SOR groups

**Fig. 4:**
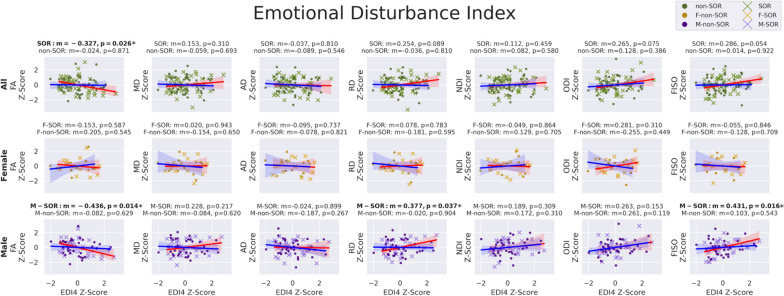
Correlation of DTI metrics (FA, MD, AD, & RD) and NODDI metrics (NDI, ODI, & FISO) in whole-brain global white matter with BASC-3 raw scores of emotional disturbance index category 4 (generalized unhappiness/withdrawal) in the SOR and non-SOR groups of school-age children. Boldface text indicates a significant correlation, with one asterisk for *p* < 0.05 uncorrected and two asterisks for FDR-corrected *p* < 0.05. SOR subjects are represented with "*x*" and non-SOR subjects with dots. All-subject comparisons are shown in green, female comparison in yellow, and male comparisons in purple. The regression line is marked red for SOR groups and blue for non-SOR groups

As hypothesized, linear regression of the seven global WM DTI and NODDI metrics versus somatization raw scores (Fig. 3) in the SOR group displays significantly negative correlation with FA and significantly positive correlations with RD and FISO. Additionally, MD shows a significant positive correlation, but this does not hold up after multiple comparison correction. Also as hypothesized, there is no significant relationship between DTI or NODDI metrics of global WM and somatization raw scores in the non-SOR control group. Sex-specific subgroup analyses show results that are directionally consistent for both boys and girls. Only the correlations involving boys with SOR initially showed significance, but this disappears after correcting for multiple comparisons. Notably, the larger sample size in males allows for better detection of trends while the study is underpowered to detect the same effect size in females. Only global WM FA manifests a strong correlation with EDI4 sum scores in the overall SOR group; however, boys with SOR also show strong correlations of EDI4 sum scores with RD and FISO (Fig. 4). These global WM associations with EDI4 sum scores in the SOR group are all directionally consistent with the ones for somatization and no such relationships are found in the non-SOR group.

### Tract-based white matter regional analysis: correlation with affective behavior

**Fig. 5:**
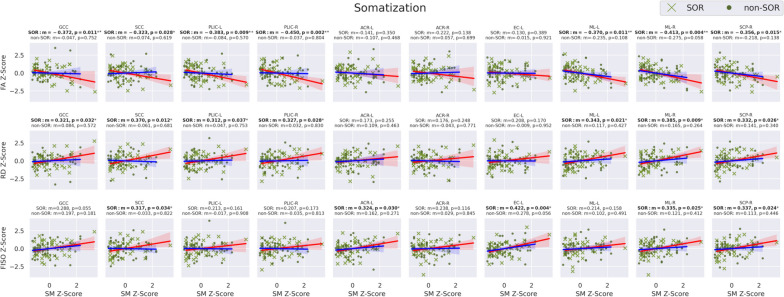
Correlation of DTI metrics: FA (*top row*) & RD (*middle row*) and NODDI metrics: FISO (*bottom row*) with BASC-3 raw scores of somatization in specific commissural, projection, association, and cerebellar & brainstem tracts in the SOR and non-SOR groups of school-age children. Boldface text indicates a significant correlation, with one asterisk for *p* < 0.05 uncorrected and two asterisks for FDR-corrected *p* < 0.05. SOR subjects are represented with "*x*" and non-SOR subjects with dots. The regression line is marked red for SOR groups and blue for non-SOR groups

**Fig. 6:**
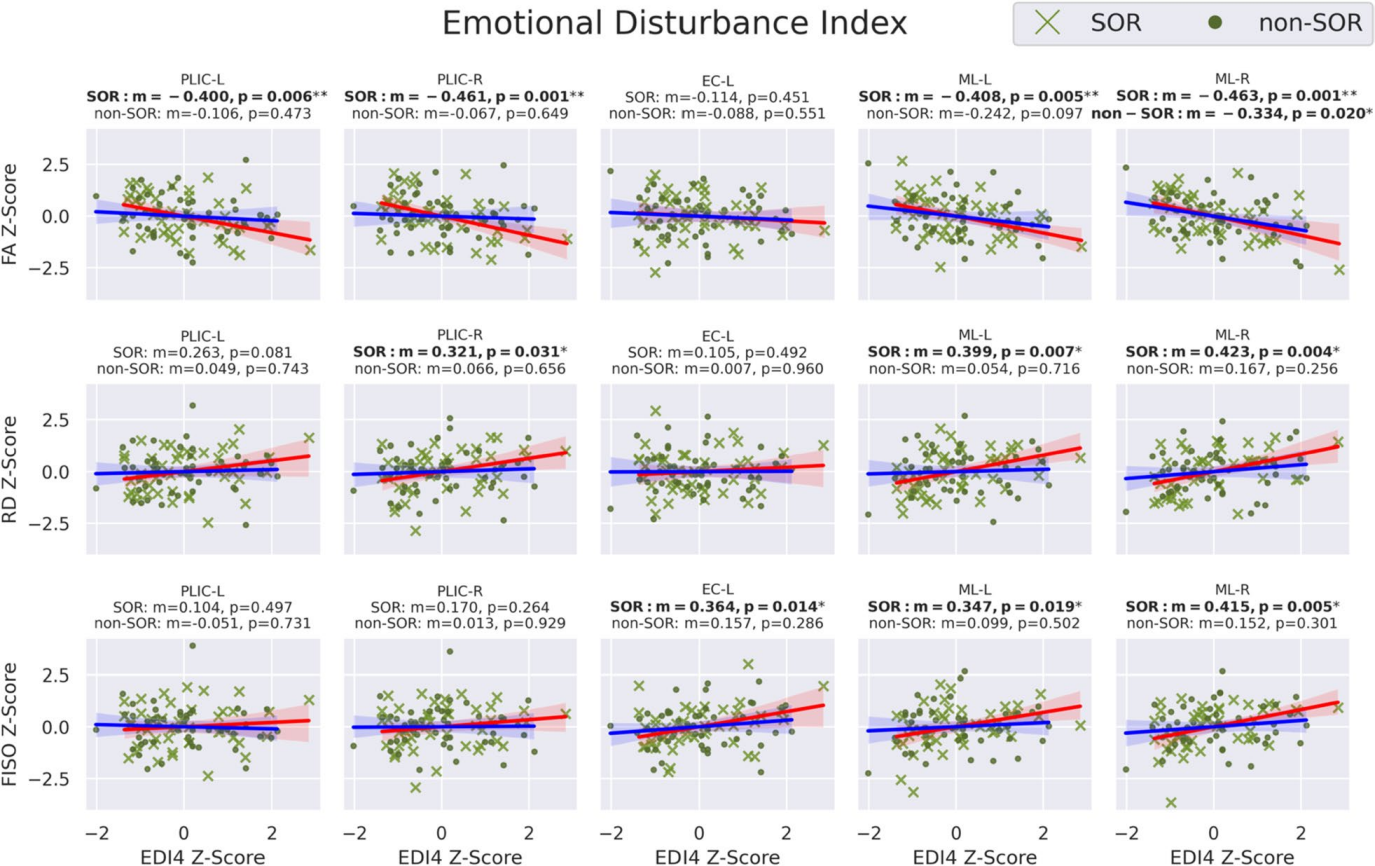
Correlation of DTI metrics: FA (*top row*) & RD (*middle row*) and NODDI metrics: FISO (*bottom row*) with BASC-3 scores of emotional disturbance category 4 (EDI4), a metric of generalized unhappiness and withdrawal, in specific commissural, projection, association, and brainstem tracts in the SOR and non-SOR groups of school-age children. Boldface text indicates a significant correlation, with one asterisk for *p* < 0.05 uncorrected and two asterisks for FDR-corrected *p* < 0.05. SOR subjects are represented with "*x*" and non-SOR subjects with dots. The regression line is marked red for SOR groups and blue for non-SOR groups

Following up on the observation of significant associations in boys of global WM FA, RD, and FISO with these BASC-3 measures of affective behavior (Figs. 3 and 4), exploratory regional WM analysis was conducted to localize the tracts most associated with somatization (Fig. 5 & Table 2) and EDI4 (Fig. 6 & Table 3). The male SOR group exhibits statistically significant negative correlations of FA with somatization in numerous major WM tracts, including interhemispheric commissural fibers of the GCC and SCC, cortical-subcortical projection fibers of the left SCR and both left and right PLIC, as well as brainstem somatosensory fibers of both left and right ML plus cerebello-cortical projection fibers of the right SCP with a strong trend also observed in the left SCP (*p* = 0.06). These negative regional FA correlations with somatization in SOR also show a positive RD correlation with somatization, except for the left SCR. Many of these same tracts also display a positive correlation of FISO with somatization in SOR.

**Table 2:** BASC-3 somatization correlations with select JHU tracts in SOR and non-SOR groups

BASC3 somatization and JHU WM correlations												
	**FA**				**RD**				**FISO**			
	SOR		Non-SOR		SOR		Non-SOR		SOR		Non-SOR	
Region	*m*	*p* value	*m*	*p* value	*m*	*p* value	*m*	*p* value	*m*	*p* value	*m*	*p* value
Corpus callosum												
GCC	− **0.372**	0.011**	− 0.047	0.752	**0.321**	0.032*	0.084	0.572	0.288	0.055	0.197	0.181
BCC	− 0.277	0.063	0.059	0.689	0.255	0.090	− 0.018	0.904	0.211	0.165	0.055	0.713
SCC	− **0.323**	0.028*	0.074	0.619	**0.370**	0.012*	− 0.061	0.681	**0.317**	0.034*	− 0.033	0.822
Internal capsule												
ALIC-L	− 0.086	0.571	− 0.051	0.733	0.032	0.833	0.061	0.678	0.126	0.408	0.102	0.490
ALIC-R	− 0.220	0.141	0.061	0.679	0.118	0.440	− 0.127	0.391	0.180	0.238	− 0.221	0.131
PLIC-L	** − 0.383**	0.009**	− 0.084	0.570	**0.312**	0.037*	0.047	0.753	0.213	0.161	− 0.017	0.908
PLIC-R	− **0.450**	0.002**	− 0.037	0.804	**0.327**	0.028*	0.032	0.830	0.207	0.173	− 0.035	0.813
Corona radiata												
ACR-L	− 0.141	0.350	− 0.107	0.468	0.173	0.255	0.109	0.463	**0.324**	0.030*	0.162	0.271
ACR-R	− 0.222	0.138	0.057	0.699	0.176	0.248	− 0.043	0.771	0.238	0.116	0.029	0.845
SCR-L	− 0.303	0.041*	0.110	0.458	0.148	0.333	− 0.100	0.497	0.190	0.212	− 0.025	0.866
SCR-R	− 0.224	0.135	0.140	0.342	0.166	0.276	− 0.151	0.304	**0.299**	0.046*	− 0.150	0.309
Association												
EC-L	− 0.130	0.389	− 0.015	0.921	0.208	0.170	− 0.009	0.952	**0.422**	0.004*	0.278	0.056
EC-R	− 0.221	0.140	0.077	0.603	0.114	0.458	− 0.066	0.654	0.178	0.242	0.041	0.781
SFO-L	− 0.015	0.919	0.051	0.732	0.131	0.393	− 0.117	0.429	0.277	0.065	− 0.124	0.401
SFO-R	0.091	0.548	− 0.034	0.818	-0.131	0.391	− 0.057	0.701	− 0.081	0.599	− 0.153	0.299
Brainstem/cerebellum												
ML-L	− **0.370**	0.011**	− 0.235	0.108	**0.343**	0.021*	0.117	0.427	0.214	0.158	0.102	0.491
ML-R	− **0.413**	0.004**	− 0.275	0.058	**0.385**	0.009*	0.165	0.264	0.335	0.025*	0.121	0.412
SCP-L	− 0.280	0.060	− 0.187	0.203	0.202	0.184	0.105	0.476	0.122	0.426	− 0.017	0.908
SCP-R	− **0.356**	0.015**	− 0.218	0.138	**0.332**	0.026*	0.141	0.340	**0.337**	0.024*	0.113	0.446

*m* represents slope of the linear regression. One asterisk indicates *p* < 0.05, two asterisks for FDR-corrected *p* < 0.05

**Table 3:** BASC-3 emotional disturbance index category 4 (generalized unhappiness/depression) correlations with select JHU tracts in SOR and non-SOR groups

BASC3 Emotional Disturbance Index:4 and JHU WM correlations												
Region	**FA**				**RD**				**FISO**			
	SOR		Non-SOR		SOR		Non-SOR		SOR		Non-SOR	
	*m*	*p* value	*m*	*p* value	*m*	*p* value	*m*	*p* value	*m*	*p* value	*m*	*p* value
Corpus callosum												
GCC	− 0.249	0.095	− 0.123	0.404	0.232	0.126	0.170	0.248	0.251	0.096	0.251	0.085
BCC	− 0.161	0.284	− 0.001	0.997	0.142	0.353	0.037	0.801	0.100	0.514	0.107	0.468
SCC	− 0.209	0.164	0.125	0.395	0.260	0.085	− 0.089	0.545	0.248	0.100	0.000	0.998
Internal capsule												
ALIC- L	− 0.001	0.994	− 0.082	0.578	− 0.050	0.743	0.073	0.621	0.057	0.712	0.071	0.629
ALIC- R	− 0.105	0.487	0.041	0.780	− 0.004	0.981	− 0.118	0.425	0.066	0.668	− 0.264	0.070
PLIC- L	− **0.400**	0.006**	− 0.106	0.473	0.263	0.081	0.049	0.743	0.104	0.497	− 0.051	0.731
PLIC-R	− **0.461**	0.001**	− 0.067	0.649	**0.321**	0.031*	0.066	0.656	0.170	0.264	0.013	0.929
Corona radiata												
ACR-L	− 0.050	0.741	− 0.146	0.321	0.038	0.805	0.170	0.247	0.271	0.072	0.205	0.163
ACR-R	− 0.183	0.223	0.007	0.963	0.103	0.500	0.002	0.989	0.223	0.141	0.029	0.846
SCR-L	− 0.152	0.314	0.037	0.801	0.016	0.915	− 0.116	0.431	0.127	0.404	− 0.112	0.447
SCR-R	− 0.151	0.316	0.003	0.981	0.068	0.659	− 0.094	0.523	0.190	0.211	− 0.163	0.268
Association												
EC-L	− 0.114	0.451	− 0.088	0.551	0.105	0.492	0.007	0.960	**0.364**	0.014*	0.157	0.286
EC-R	− 0.180	0.231	0.005	0.971	− 0.005	0.976	− 0.045	0.759	0.046	0.766	− 0.080	0.587
SFO-L	0.098	0.515	0.089	0.548	− 0.052	0.735	− 0.196	0.182	0.178	0.242	− 0.200	0.172
SFO-R	0.168	0.265	− 0.032	0.829	− 0.230	0.128	− 0.098	0.509	− 0.176	0.247	− 0.244	0.095
Brainstem/cerebellum												
ML-L	− **0.408**	0.005**	− 0.242	0.097	**0.399**	0.007*	0.054	0.716	**0.347**	0.019*	0.099	0.502
ML-R	− **0.463**	0.001**	− **0.334**	0.020*	**0.423**	0.004*	0.167	0.256	**0.415**	0.005*	0.152	0.301
SCP-L	− 0.193	0.200	− 0.150	0.310	0.049	0.747	0.051	0.731	− 0.009	0.952	− 0.003	0.984
SCP-R	− 0.210	0.160	− 0.138	0.351	0.121	0.427	0.062	0.673	0.161	0.290	0.097	0.511

*m* represents slope of the linear regression. One asterisk indicates *p* < 0.05, two asterisks for FDR-corrected *p* < 0.05

Furthermore, some tracts with no significant FA or RD association with somatization in boys with SOR do show such a relationship between FISO and somatization. These tracts are the left ACR and the left EC and both also display positive correlations. Notably, there were no significant regional WM correlations of FA, RD, or FISO with somatization in the non-SOR group, whether males or females.

Of the eleven investigated tracts, the bilateral ML and the bilateral PLIC evince similar relationships of FA, RD, and/or FISO with EDI4 as they do with somatization in boys with SOR (Fig. 6 & Table 3). Moreover, the left EC also manifests a positive correlation of FISO with EDI4 as it does with somatization. None of the tracts have a significant microstructural correlation with EDI4 in the non-SOR group, whether male or female, except for FA of the right ML in non-SOR boys, which shows a negative correlation that is not as strong or as statistically significant as in the boys with SOR.

The regional exploratory analysis of JHU WM tracts was completed post hoc to determine which tracts were most responsible for the global metric results; given the exploratory nature of the analysis, significance was reported both with and without multiple comparisons correction. The regional, global, and JHU analyses for the somatization correlations were ran without the male SOR subject with the Somatization z-score greater than three (Supplementary Fig. 2 & Supplementary Fig. 3). In both the global and JHU correlations for the all and Male SOR and non-SOR comparisons where this subject was removed, most significant findings remain unchanged and those that lost significance remained strong trends or with similar slope directionality.

## Discussion

### White matter microstructural alterations in broadly defined sensory processing dysfunction

Consistent with the premise of reduced white matter microstructural integrity of specific neural networks, we have previously reported that children born prematurely show increased sensory processing differences, particularly in the auditory domain, and that these children are also known to have brain injury that is “regional,” in the posterior periventricular white matter [2, 3]. This regional predilection is thought to be related to vulnerability of oligodendrocyte precursors—thus “at-risk” territory [4]. Furthermore, children with agenesis of the corpus callosum, a syndrome of hemispheric disconnection, also show differences in sensory processing [5, 6]. However, in the extant literature, sensory dysfunction in children with genetic and injury-based conditions is approached with a broad sensory framework that does not answer the question of whether SOR results from a regional disruption and/or plasticity of dedicated neuronal networks. Without this information, it becomes impossible to quantify network neuroplasticity with targeted treatment interventions. However, posterior periventricular WM contains particularly broad and dense connectivity within the cerebral cortical connectome and can therefore affect many different cognitive and behavioral domains [56].

### Biophysical modeling of diffusion MRI advances understanding of white matter microstructure

Although DTI is a useful tool for studying brain development, it represents only a basic statistical description of water diffusion within a voxel from images typically acquired at a single relatively low diffusion-weighting factor (*b* value) representing only a single spherical shell in q-space. The assumption of Gaussian diffusion that underpins the DTI model breaks down at *b* values in excess of 1000 s/mm^2^, whereas the investigation of restricted and strongly hindered diffusion, such as within the intracellular space, requires higher diffusion-weighting factors. Therefore, common DTI measures, specifically FA, MD, AD, and RD, lack the specificity to differentiate between intracellular and extracellular disruption [57, 58].

In contrast to DTI, NODDI is a multi-compartment biophysical model of brain microstructure that computes the non-collinear properties of neurite orientation dispersion index (ODI) and neurite density index (NDI) within each imaging voxel. NODDI employs a tissue model that distinguishes three types of microstructural environment: (1) restricted intra-cellular compartment modeled with orientation dispersion using a Watson distribution, (2) extra-cellular compartment with Gaussian anisotropically hindered diffusion, and (3) cerebrospinal fluid (CSF) compartment with freely isotropic diffusion [36, 37]. One advantage of NODDI over previous biophysical diffusion models is that the multi-shell HARDI imaging data is within the current MR scanner’s hardware, pulse sequence, and acquisition time constraints for clinical studies. Additionally, free water diffusion is isolated into a separate biophysical compartment (FISO); therefore, CSF partial volume averaging does not contaminate estimates of tissue microstructure as it can with DTI.

### Global white matter microstructure in children with SOR and its association with behavior

In this study, we provide evidence for our a priori hypotheses that microstructural WM differences in SOR are associated with variation in affective behavior, particularly somatization and the consequent emotional disturbance characterized by generalized unhappiness and withdrawal. This relationship is strong in boys with SOR, but not in girls. However, the sample size of girls in our cohort is too small to detect small to moderate effect sizes, given that fewer females are affected by SOR or other forms of SPD than males. No significant correlation of global WM microstructure with somatization or generalized depression is observed in children with neurodevelopmental concerns but not SOR specifically, whether boys or girls. Although statistically significant differences in global WM DTI and NODDI metrics were found between girls with versus without SOR, these findings need replication in larger studies given the relatively small female sample size of the present investigation.

Boys with SOR who have greater global WM microstructural integrity on DTI in the form of higher FA and lower RD are relatively protected against somatization and emotional disturbance, whereas those with reduced FA and elevated RD have greater susceptibility. NODDI analysis shows that these differences in WM microstructural intergity are primarily due to differences in free water content as measured by FISO. Free water has a much higher diffusivity rate than brain tissue and essentially zero diffusion anisotropy. Therefore, all else being equal, high FISO necessarily results in low FA and high RD, and is thus a marker of poor microstructural integrity. There are several possible etiologies for elevated FISO, including neuroinflammation with vasogenic edema as well as expansion of the CSF-filled perivascular spaces. These two processes can be interrelated in dysfunction of the glympatic system [59].

A diffuse axonal disconnection that affects global white matter and causes general intellectual disability is not present in our sample of children with neurodevelopmental concerns, including those with SOR. There are no statistically significant differences of global WM DTI and NODDI metrics between boys with SOR and boys in the non-SOR group. Although some such DTI and NODDI differences are seen in girls with SOR vs non-SOR, these are based on a small sample size and will need to be confirmed in a larger investigation. Notably, no group mean differences in somatization or EDI4 are observed between the SOR and non-SOR groups, especially not in boys. What differs between SOR and non-SOR is the dependence of somatization and emotional disturbance on white matter tract microstructural integrity in the former but not the latter cohort. This represents an objective brain-based correlate for SOR and behavior, when SOR status is determined from a modern structured direct clinical assessment method, the SP3D:A.

### Regional white matter microstructure in children with SOR and its association with behavior

The results of the exploratory tract ROI analysis showed that, for boys with SOR, there are significant WM microstructural correlations with somatization in major commissural, projection, association, and brainstem/cerebellar pathways. The strongest of these associations are found for FA in cortical-subcortical projection pathways of the bilateral PLIC and bilateral ML, all of which survived correction for multiple tract-wise comparisons. Similarly, the bilateral PLIC and bilateral ML are also the tracts most correlated with generalized unhappiness/withdrawal in SOR males, remaining significant for FA after multiple comparison correction. All of these regional correlations of FA, RD, and FISO are directionally consistent with what is found for global WM in boys with SOR. No significant relationship of regional WM microstructure with somatization is found in boys with neurodevelopmental concerns but not SOR. The right ML does show a significant correlation of FA with emotional disturbance in males of the non-SOR group, although its strength is less than that of the SOR group and does not survive multiple comparison correction.

The medial lemnisci are well known as the primary somatosensory tracts of the brainstem, containing axonal fibers transmitting information about fine touch and two-point discrimination as well as conscious proprioception and vibration. This second-order somatosensory pathway extends from the medulla to the ventral posterolateral (VPL) nucleus of the thalamus, bridging the first-order pathway in the dorsal column of the spinal cord and the third-order pathway from the VPL to the primary somatosensory cortex. The third-order somatosensory pathway extends into the posterior limb of the internal capsule, a structure that also contains auditory pathway fibers extending via the lateral lemniscus from the inferior colliculus to the temporal lobe [60]. Given that the SOR cohort in this study consists almost entirely of individuals with auditory and/or tactile over-responsivity, it is not at all surprising that microstructural WM integrity of the ML and PLIC would be important for behavioral sequelae in this condition. Posterior WM tracts have previously been implicated in broadly defined SPD, with Chang et al. [25] demonstrating significant correlations of FA in the PLIC with caregiver assessments of auditory and tactile dysfunction using the sensory profile as well as with objective testing of auditory and tactile function using the Acoustic Index of the Differential Screening Test for Processing (DSTP) and using graphesthesia, respectively. However, to our knowledge, WM microstructure of the ML has not previously been investigated in children with SPD.

Besides the ML and PLIC, several commissural, association, projection, and cerebellar tracts exhibit significant correlations with somatization that require confirmation in future hypothesis-driven studies. One such tract, the splenium of the corpus callosum, has been well recognized to have low FA and high RD in children with SPD compared to matched typically developing children (TDC) [32], that are related to auditory and tactile behavior and function [25]. The major cerebellar outflow tract (SCP) and inflow tract (MCP) are known to have reduced FA and increased RD in SPD compared to TDC as well as association with auditory behavior, multisensory integration, and attentional function [33]. Beyond these posterior cerebral and hindbrain tracts, our study also implicates the prefrontal WM pathways of the genu of the corpus callosum and left anterior corona radiata as relevant to somatization in boys with SOR. These two tracts, responsible for prefrontal interhemispheric communication and for prefrontal to subcortical connectivity respectively, are associated with affective behavior such as depression [61], but have not been previously investigated in the context of SPD and of SOR in particular.

### Comparison to prior neuroimaging studies of affective behavior

There is a dearth of prior research in children on white matter microstructural correlates of somatization, and to our knowledge, none is related to sensory processing dysfunction. However, a recent gray matter morphometry study of adults with somatic symptoms disorder reveals that cerebellar gray matter volumes are negatively correlated with somatization and that cortico-cortical and basal gangla to cerebellar structural covariance is also associated with somatization [62]. This is in general agreement with our findings of a widespread network of white matter pathways related to somatization in boys with SOR, including cortico-cortical association tracts, cortico-subcortical projection tracts, and cerebellar pathways. Alterations of corticothalamic functional connectivity to somatosensory, auditory, and visual cortex have also been found in somatization disorder in adults using resting state functional MRI (fMRI) [63], which thereby implicates the PLIC and other posterior sensory WM tracts.

In the related condition of internalizing problems, an early pilot DTI study of school-age children born preterm using TBSS found that the global WM FA is inversely correlated with internalizing behavior measured with the Child Behavior Checklist (CBCL) parent report form [64]. A regional analysis showed significant correlations with several WM tracts, most notably the forceps minor and major, which contain commissural fibers of the genu and splenium of the corpus callosum, respectively. Given the aformentioned high rate of SPD in children born preterm [2, 3], these WM microstructural changes might have been related to sensory processing dysfunction; however, no sensory behavioral testing was performed as part of the investigation. Our findings suggest a role for sensory over-responsivity in the relationship with FA and that this inverse association is driven by opposing changes in RD, with both the FA and RD alterations primarily the result of variation in the free water content of the affected white matter. A more recent DTI study of internalizing behavior using the BASC-2 rating scale in typically developing children indicates a correlation with MD of the bilateral cingulum; however, children with neurodevelopmental concerns were not included and there was no assessment of sensory behavior [65].

A recent DTI and NODDI voxelwise analysis of SOR in young adults with ASD compared to TDC controls discovered elevated NDI in the right superior temporal gyrus in ASD versus TDC as well as a positive correlation of NDI with adverse childhood experiences (ACE) in the ASD group but not the TDC controls [66]. Greater ACE was also related to SOR severity. This study is concordant with our findings in that it also indicates microstructural neuroplasticity associated with SOR. The determination of SOR status was from the adolescent/adult sensory profile, a self-report questionnaire, rather than the SP3D:A clinical assessment used in our work. Also, we excluded both ASD and TDC in our research, focusing on children with neurodevelopmental concerns but not ASD that represent the largest population referred for clinical evalution.

We also observed that, like somatization, reduced FA, elevated RD and/or elevated FISO in the bilateral PLIC and bilateral ML, as well as the left EC, are associated with the BASC-3 emotional disturbance index category 4 (generalized unhappiness and withdrawal) score in boys with SOR. Low FA and high RD of white matter have previously been found in adolescent and adult major depressive disorder [67, 68]. As with the global WM findings related to EDI4, our NODDI results suggest that, at least for those with SOR, these DTI alterations are driven by changes in free water content of these affected tracts. Recent DTI and NODDI research on school-age children with broadly defined SPD found that, in boys especially, those with comorbid ADHD have lower FA in the internal capsule and splenium of the corpus callosum than those without ADHD [69]. However, those attention- and impulsivity-related changes of WM FA are due to lower NDI and not somatization- and depression-related higher FISO like in our SOR group.

### Limitations and future directions

Our results support the concept that compensatory neuroplasticity of white matter microstructure may influence affective behavior in children with SOR in a way that is not seen in other children with neurodevelopmental concerns. However, alternate interpretations of these cross-sectional data are possible, such as innate differences in white matter microstructure that are correlated with emotional responses in SOR that do not change appreciably during brain development. Hence, the observations reported herein require further investigation in a larger more diverse cohort with a broader array of cognitive and behavioral assessments, including multi-year follow-up of psychological, health, educational, social, and economic outcomes to investigate the long-term neurodevelopmental trajectory of SOR. Extending the age range studied to younger chidren would improve the ability to chart longitudinal changes during development [70, 71]; however, practical difficulties remain in performing advanced imaging of unsedated young chidren. Multimodal imaging incorporating fMRI can directly interrogate the activity and functional connectivity of gray matter to incorporate with microstructural and structural connectivity data from dMRI. The validation of neural correlates of SOR that are linked to affective behavior through these future studies would pave the way toward objective brain-based biomarker development that can better stratify risk for adverse mental health outcomes in children for patient selection in clinical trials of cognitive, behavioral, occupational, and pharmacological therapies and also for monitoring treatment efficacy as intermediate endpoints. This is a major and growing unmet public health need given the explosion of depression, loneliness/withdrawal, and suicidality among youth in recent years [72].

## Conclusions

Sensory over-responsivity in school-age children, determined from the SP3D:A structured comprehensive clinical assessment, is characterized by variation in global white matter microstructural integrity that explains differences in affective behavior, particularly somatization and emotional disturbance in the form of depression and withdrawal. Reduced fractional anisotropy and elevated radial diffusivity on DTI are associated with maladaptive behavior in SOR, most likely caused by elevated free water content in white matter on NODDI analysis. Second-order and third-order sensory pathways of the medial lemniscus and posterior limb of internal capsule, respectively, are the most affected white matter tracts, although a widespread network of other cerebral, cerebellar, and brainstem tracts are also involved. Boys with SOR exhibit the white matter association with affective behavior more strongly than girls; however, given the much lower rate of SOR and SPD more broadly in females than males, the sample size of girls is too small for definitive conclusions. These findings suggest that comprehensive evaluation of sensory behavior may lead to more specific neuroimaging biomarkers for personalized healthcare to avoid adverse intellectual, social, and mental health outcomes in children.

### Supplementary Information

**Additional file 1: Fig. S1.** ESSENSE-Q-REV parent report form. **Fig. S2**. Correlation of DTI metrics (FA, MD, AD, & RD) and NODDI metrics (NDI, ODI, & FISO) in whole-brain global white matter with BASC-3 raw scores of somatization in the SOR and non-SOR groups of school-age children, excluding the male SOR subject with the SM score outlier. Metric “m” is the slope of the linear regression and color shading around the regression line represents the 95% confidence interval. Boldface text indicates a significant correlation, with one asterisk for *p*<0.05 uncorrected and two asterisks for FDR-corrected *p*<0.05. SOR subjects are marked with dots and non-SOR subjects with “x”. The all-subject comparisons are shown in green, female comparison in yellow and male comparisons in purple. The red line is the regression line for the SOR groups and the blue for the regression line of the non-SOR groups. **Fig. S3**. Correlation of DTI metrics: FA (*top row*) & RD (*middle row*) and NODDI metrics: FISO (*bottom row*) with BASC-3 raw scores of somatization in specific commissural, projection, association, and cerebellar & brainstem tracts in the SOR and non-SOR groups of school-age children, excluding the male SOR subject with the SM score outlier. Metric “m” is the slope of the linear regression and color shading around the regression line represents the 95% confidence interval. Boldface text indicates a significant correlation, with one asterisk for *p*<0.05 uncorrected and two asterisks for FDR-corrected *p*<0.05. SOR subjects are marked with dots and non-SOR subjects with “x”. The all-subject comparisons are shown in green, female comparison in yellow and male comparisons in purple. The red line is the regression line for the SOR groups and the blue for the regression line of the non-SOR groups.

## Data Availability

The datasets presented in this study can be found in online repositories. The names of the repository/repositories and accession number(s) can be found at the NIH Data Archive (https://nda.nih.gov) Accession Number:4095004.

## Abbreviations

ACR: Anterior corona radiata
AD: Axial diffusivity
ADHD: Attention deficit/hyperactivity
ADOS-2: Autism Diagnostic Observation Schedule, Second Edition
ALIC: Anterior limb part of internal capsule
AMICO: Accelerated microstructure imaging via convex optimization
AOR: Auditory over-responsivity
ASD: Autism spectrum disorder
BCC: Body of corpus callosum
CGC: Cingulum (cingulate gyrus)
CGH: Hippocampal cingulate bundle
CGH: Cingulum (hippocampus)
CP: Cerebral peduncle
CSF: Cerebrospinal fluid
CST: Corticospinal tract
dMRI: Diffusion MRI
DSM: Diagnostic and statistical manual
DTI: Diffusion tensor imaging
EC: External capsule
ER: Emotional resilience
ESSENCE: Early Symptomatic Syndromes Eliciting Neurodevelopmental Clinical Examinations
F-AOR: Females with auditory over-responsivity
F-non-SOR: Females without sensory over-responsivity
F-SOR: Females with sensory over-responsivity
FA: Fractional anisotropy
FDR: False discovery rate
FWE: Family-wise error
FISO: Free water fraction
FSL: FMRIB Software Library
GCC: Genu of corpus callosum
HARDI: High angular resolution diffusion imaging
ICP: Inferior cerebellar peduncle
JHU: John’s Hopkin’s University
L: Left
M-AOR: Males with auditory over-responsivity
M-non-SOR: Males without sensory over-responsivity
SOR: Males with sensory over-responsivity
MCP: Middle cerebellar peduncle
MD: Mean diffusivity
ML: Medial lemniscus
MRI: Magnetic resonance imaging
ND: Neuropsychiatric distress
NDI: Neurite density index
NODDI: Neurite orientation dispersion and density imaging
non-SOR: Without sensory over-responsivity
ODI: Orientation dispersion index
PCR: Posterior corona radiata
PCT: Pontine crossing tract
PLIC: Posterior limb part of internal capsule
PTR: Posterior thalamic radiation
R: Right
RD: Radial diffusivity
RLIC: Retrolenticular part of internal capsule
ROI: Region of interest
SCC: Splenium of corpus callosum
SCP: Superior cerebellar peduncle
SCQ: Social Communication Questionnaire
SCR: Superior corona radiata
SFO: Superior fronto-occipital fasciculus
SLF: Superior longitudinal fasciculus
SOR: Sensory over responsivity
SP3D:A: Sensory Processing 3 Dimensions: Assessment
SPD: Sensory processing dysfunction
SS: Sagittal stratum
TBSS: Tract-based spatial statistics
TFCE: Threshold-free cluster enhancement
TOR: Tactile over-responsivity
UNC: Uncinate fasciculus
VOR: Visual over-responsivity
WM: White matter
