# Supplementary material for: White matter microstructure of children with sensory over-responsivity is associated with affective behavior
Source: J Neurodev Disord. 2024 Jan 2;16:1. doi: 10.1186/s11689-023-09513-w (PMC10759342; doi:10.1186/s11689-023-09513-w)
Supplement: Supplementary file 1 — Additional file 1: Fig. S1. ESSENSE-Q-REV parent report form. Fig. S2. Correlation of DTI metrics (FA, MD, AD, & RD) and NODDI metrics (NDI, ODI, & FISO) in whole-brain global white matter with BASC-3 raw scores of somatization in the SOR and non-SOR groups of school-age children, excluding the male SOR subject with the SM score outlier. Metric “m” is the slope of the linear regression and color shading around the regression line represents the 95% confidence interval. Boldface text indicates a significant correlation, with one asterisk for p<0.05 uncorrected and two asterisks for FDR-corrected p<0.05. SOR subjects are marked with dots and non-SOR subjects with “x”. The all-subject comparisons are shown in green, female comparison in yellow and male comparisons in purple. The red line is the regression line for the SOR groups and the blue for the regression line of the non-SOR groups. Fig. S3. Correlation of DTI metrics: FA (top row) & RD (middle row) and NODDI metrics: FISO (bottom row) with BASC-3 raw scores of somatization in specific commissural, projection, association, and cerebellar & brainstem tracts in the SOR and non-SOR groups of school-age children, excluding the male SOR subject with the SM score outlier. Metric “m” is the slope of the linear regression and color shading around the regression line represents the 95% confidence interval. Boldface text indicates a significant correlation, with one asterisk for p<0.05 uncorrected and two asterisks for FDR-corrected p<0.05. SOR subjects are marked with dots and non-SOR subjects with “x”. The all-subject comparisons are shown in green, female comparison in yellow and male comparisons in purple. The red line is the regression line for the SOR groups and the blue for the regression line of the non-SOR groups. [file 11689_2023_9513_MOESM1_ESM.docx]

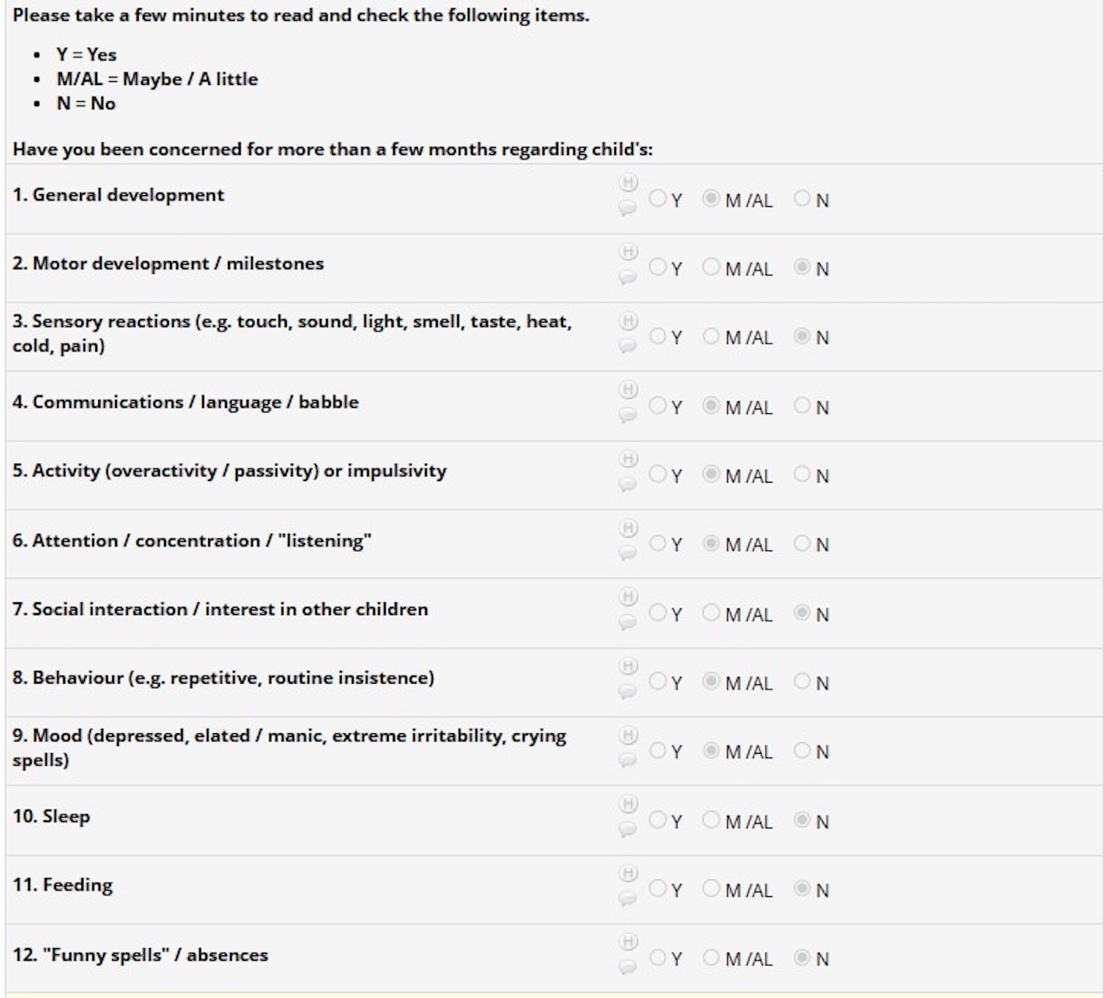


**Supplementary Figure 1.** ESSENSE-Q-REV parent report form.


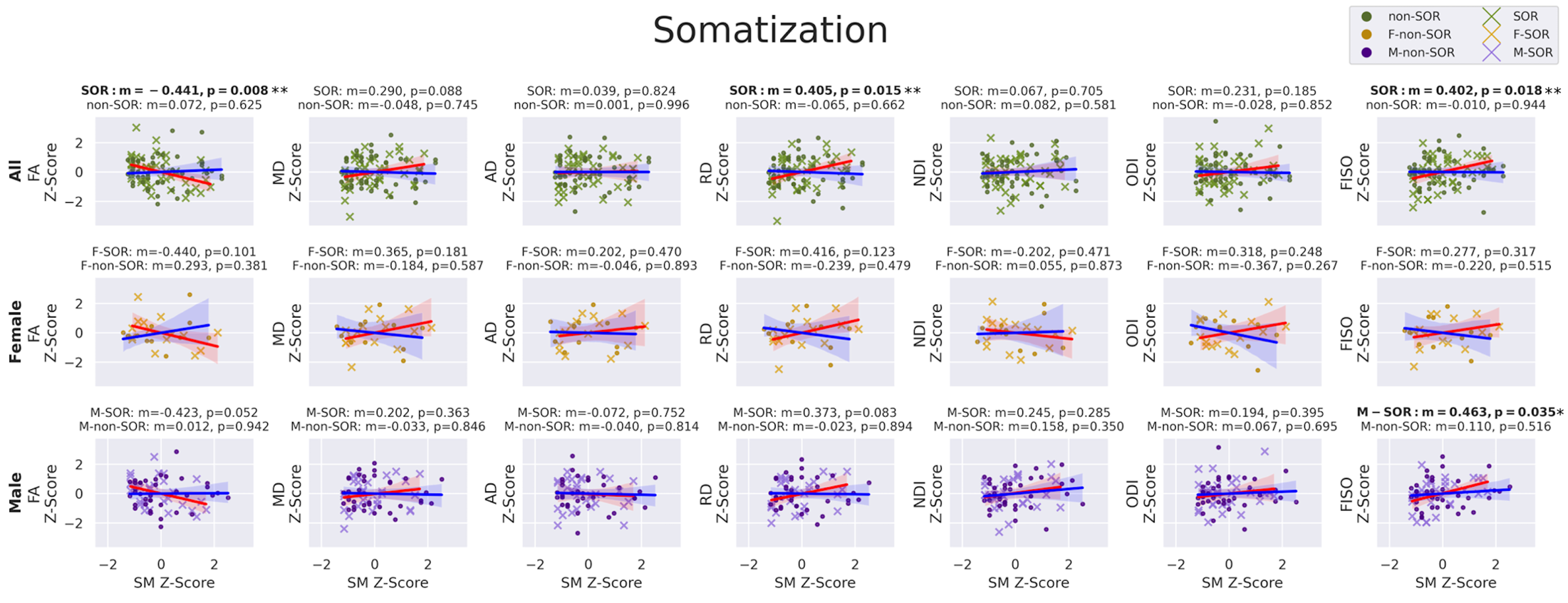


**Supplementary Figure 2**. Correlation of DTI metrics (FA, MD, AD, & RD) and NODDI metrics (NDI, ODI, & FISO) in whole-brain global white matter with BASC-3 raw scores of somatization in the SOR and non-SOR groups of school-age children, excluding the male SOR subject with the SM score outlier. Metric “m” is the slope of the linear regression and color shading around the regression line represents the 95% confidence interval. Boldface text indicates a significant correlation, with one asterisk for p<0.05 uncorrected and two asterisks for FDR-corrected p<0.05. SOR subjects are marked with dots and non-SOR subjects with “x”. The all-subject comparisons are shown in green, female comparison in yellow and male comparisons in purple. The red line is the regression line for the SOR groups and the blue for the regression line of the non-SOR groups.

**
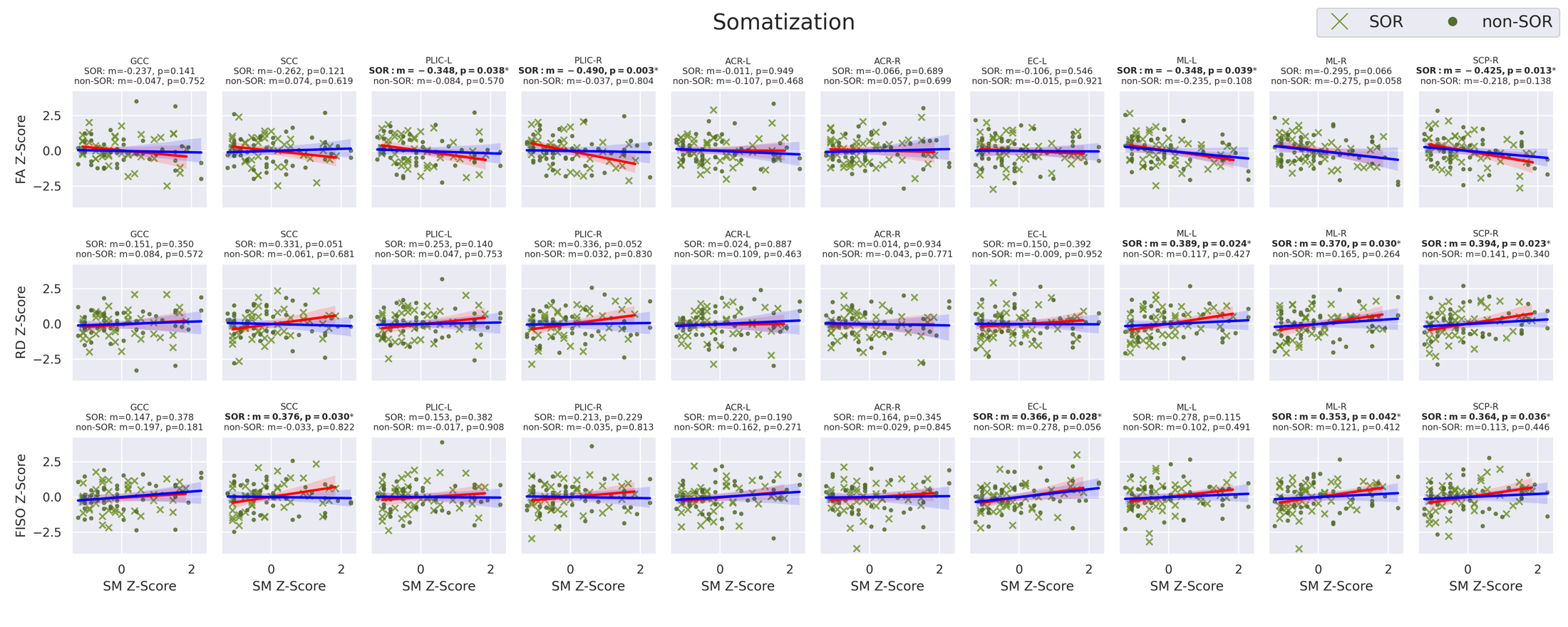
Supplementary Figure 3**. Correlation of DTI metrics: FA (*top row*) & RD (*middle row*) and NODDI metrics: FISO (*bottom row*) with BASC-3 raw scores of somatization in specific commissural, projection, association, and cerebellar & brainstem tracts in the SOR and non-SOR groups of school-age children, excluding the male SOR subject with the SM score outlier. Metric “m” is the slope of the linear regression and color shading around the regression line represents the 95% confidence interval. Boldface text indicates a significant correlation, with one asterisk for p<0.05 uncorrected and two asterisks for FDR-corrected p<0.05. SOR subjects are marked with dots and non-SOR subjects with “x”. The all-subject comparisons are shown in green, female comparison in yellow and male comparisons in purple. The red line is the regression line for the SOR groups and the blue for the regression line of the non-SOR groups.
